# Chemotherapy and Oncolytic Virotherapy: Advanced Tactics in the War against Cancer

**DOI:** 10.3389/fonc.2014.00145

**Published:** 2014-06-11

**Authors:** Andrew Nguyen, Louisa Ho, Yonghong Wan

**Affiliations:** ^1^Department of Pathology and Molecular Medicine, McMaster Immunology Research Centre, McMaster University, Hamilton, ON, Canada

**Keywords:** oncolytic virotherapy, cancer immunotherapy, cancer vaccines, combination therapy, drug therapy, combination, oncolytic viruses

## Abstract

Cancer is a traitorous archenemy that threatens our survival. Its ability to evade detection and adapt to various cancer therapies means that it is a moving target that becomes increasingly difficult to attack. Through technological advancements, we have developed sophisticated weapons to fight off tumor growth and invasion. However, if we are to stand a chance in this war against cancer, advanced tactics will be required to maximize the use of our available resources. Oncolytic viruses (OVs) are multi-functional cancer-fighters that can be engineered to suit many different strategies; in particular, their retooling can facilitate increased capacity for direct tumor killing (oncolytic virotherapy) and elicit adaptive antitumor immune responses (oncolytic immunotherapy). However, administration of these modified OVs alone, rarely induces successful regression of established tumors. This may be attributed to host antiviral immunity that acts to eliminate viral particles, as well as the capacity for tumors to adapt to therapeutic selective pressure. It has been shown that various chemotherapeutic drugs with distinct functional properties can potentiate the antitumor efficacy of OVs. In this review, we summarize the chemotherapeutic combinatorial strategies used to optimize virally induced destruction of tumors. With a particular focus on pharmaceutical immunomodulators, we discuss how specific therapeutic contexts may alter the effects of these synergistic combinations and their implications for future clinical use.

*Do not repeat the tactics, which have gained you one victory, but let your methods be regulated by the infinite variety of circumstances*.

–Sun Tzu, The Art of War

## Introduction

Oncolytic viruses (OVs) can selectively infect, replicate in, and kill tumor cells with minimal impact on normal tissue. These tumor-specific properties, called oncotropism, is dependent on the expression of surface receptors that allow viral binding and entry, as well as, the permissiveness of the tumor cell toward viral replication. Genetic manipulation of the viral genome aims to improve the inherent therapeutic value of OVs by enhancing their capacity for targeted tumor killing (1, 2). Through transgene insertion, OVs can serve as directed gene-delivery vehicles, and thus accommodate a diverse array of therapeutic strategies. Arming OVs with additional weaponry, such as pro-apoptotic genes, tumor suppressors, or genes stimulating antitumor immunity, can enhance their killing capacity. With a broad arsenal, modified-OVs have the potential to target a wide spectrum of different cancer types. However, administration of OVs as a monotherapy has demonstrated varying degrees of success in clinical trials (3–5). This is likely due to host antiviral immune-mediated mechanisms that limit OV dissemination and promotes pre-mature viral clearance. Over an extended period, selective pressure on heterogeneous tumor populations can also lead to therapeutic resistance to OVs via receptor loss or mutation of essential signaling pathways required for viral replication (6). To overcome these barriers, many clinically established and novel chemotherapeutics have been used in combination with oncolytic virotherapy, showing synergistic effects that potentiate tumor killing (7–9). In this review, we summarize how immunomodulatory chemotherapeutic combinatorial strategies have been used to optimize virally induced destruction of tumors and discuss their implications for future directions and clinical use.

## Mechanisms of Oncolytic Viruses

### Tumor tropism and oncolysis

The oncotropism of viruses is guided by cell surface receptors that enable viral binding and entry, and the permissiveness of the infected cell to viral replication. Surface receptors that are recognized by different types of viruses can be specific to neoplastic cells. These viruses target receptors characteristic of malignant phenotypes, such as Poliovirus that binds CD-155 that is almost exclusively present in high grade glioma cells (10, 11), and Sindbis virus that recognizes high-affinity laminin receptor overexpressed in many cancers (12). Other viruses, such as vesicular stomatitis virus (VSV) exhibit a remarkably robust and pantropic selectivity by binding to the ubiquitously expressed LDL receptor (13). Therefore, instead of relying on receptor specificity, tumor tropism of VSV is dependent on the permissiveness of malignant cells to viral infection. VSV belongs to a class of interferon (IFN)-sensitive viruses, which preferentially infects tissues exhibiting reduced or absent IFN responsiveness (14–17). This is a typical feature of tumors, which often acquire defects in pathways involved in innate antiviral immunity, such as the IFN pathway, as a mechanism for immune escape. In fact, many of the biological pathways altered by viral infection are similar to cellular changes acquired during carcinogenesis. For instance, mutated oncogenes such as BRAF or Cyclin A, increases the infectivity of VSV and parvovirus, respectively (18, 19). As well, impaired apoptotic ability typically observed in neoplastic cells provides an opportunity for OVs to enhance their replicative capacity (20).

Selective retargeting of viruses to tumor cells can also be generated in viruses without innate oncolytic abilities. Adenovirus (Ad)-based vectors are a good demonstration of this approach, since they possess a wide tropism, but a lytic life cycle that can be exploited for oncolytic virotherapy (21). One method to restrict viral replication to tumor cells is the modification of E1A and E1B genes that results in conditionally replicative Ad. As a result, selective replication occurs in cells defective in p53 or Rb tumor suppressor pathways; a characteristic observed in 50% of human cancers (22). Alternatively, various transductional retargeting strategies exist that largely involve fusing tumor targeting ligands to the Ad fiber knob domain, summarized in Ref. (23).

Viral oncolysis directly destroys tumor cells through either their lytic replication cycle or the expression of endogenous cytotoxic gene products (24). To further enhance their oncolytic effects, transgenes encoding pro-apoptotic proteins are inserted into OVs to subvert cell death machinery. These proteins include various death-inducing ligands such as TNF-related apoptosis-inducing ligand (TRAIL) (25, 26), Fas ligand (FasL) (27), and tumor suppressor genes (e.g., p53, p16) (28, 29). Alternatively, small hairpin RNA targeting factors can be inserted to silence genes involved in cell survival or proliferation, including hTERT and ki67 (30) or MYCN oncogene (31). Oncolytic viral infection can also induce autophagy, a conserved catabolic process crucial in maintaining cellular homeostasis (32). Cellular autophagy machinery is disrupted by certain viruses to facilitate its own replication (33, 34) and enhance oncolysis (35, 36). By engineering viruses to express autophagy-inducing genes, such as Beclin-1 (37) and mTOR pathway regulators (38, 39), improved therapeutic outcomes can be achieved. This approach may be particularly useful for treating apoptosis-resistant types of cancer, thus warranting further development toward clinical application. Lastly, some OVs can exert indirect mechanisms of tumor killing, including tumor vascular shutdown (40, 41) and the induction of antitumor immune responses, the latter of which is described in further detail in the following section.

### Induction of antitumor immune responses

The various mechanisms through which OVs are capable of lysing cancer cells result in the release of tumor associated antigens (TAAs), proinflammatory cytokines, chemokines, and other danger signals, which facilitates immune cell recruitment and activation within tumors. In particular, activation and maturation of dendritic cells (DCs) and other antigen presenting cells (APCs) allow for efficient cross-presentation to T cells, and subsequent initiation of antitumor and antiviral immune responses (42, 43). However, OVs induce only weak tumor-specific immune responses, due to premature viral clearance and immunosuppressive regulatory factors within the tumor.

To potentiate their immunogenic effects, genetic engineering strategies have been used to encode OVs with various cytokines, immunomodulators, and TAAs (44, 45). Evaluation of the antitumor efficacy of OVs expressing cytokines, such as IL-12, IL-2, IL-4, IL-18, IL-24, and TNFα, has shown improved therapeutic effects (46–49). One of the most promising cytokines tested within the OV platform to date, is the granulocyte-macrophage colony-stimulating factor (GM-CSF), which promotes DC maturation and induces tumor antigen-specific cytotoxic T cells. Three major viral vectors, Ad, VV, and HSV, armed with GM-CSF have been demonstrated to enhance antitumor immunity and cytotoxicity in several clinical trials (50–57). In particular, Talimogene laherparepvec (T-VEC), a GM-CSF-expressing oHSV-1 that has recently completed phase III trials in melanoma and head and neck cancer, are the first to demonstrate efficacy of OV immunotherapy, with an approximately 30% response rate against systemic disease, following local injection into accessible tumors (52, 53). Similar to GM-CSF, Fms-like tyrosine kinase-3 ligand (FLT3L) is a potent growth factor capable of recruiting and expanding DCs *in vivo* (58). OVs expressing FLT3L trigger DC and T cell infiltration into the tumor and enhance both antitumoral and antiviral immune responses (42, 59, 60), implicating potential benefits of using FLT3L as an adjuvant to cancer vaccination. Another strategy to boost the antitumor response involves genetically engineering OVs to express inflammatory chemokines, and thus increasing the number of tumor-infiltrating immune cells. Expression of CCL5, CCL3, and CCL19 by OVs enhances chemotaxis of immune cells within the tumor and improves overall therapeutic benefits *in vivo* (61–64). Interestingly, distinct effects on virus activity were also observed, in which VV expressing CCL5 or CCL19 resulted in increased persistence within the tumor and more rapid clearance from non-tumor tissues, respectively (61, 65, 66). Finally, cross-presentation of TAA to T cells through DC activation can also be achieved by arming OVs with co-stimulatory molecules such as CD40L (67, 68) and heat shock proteins (69).

A more direct approach to engage antigen-specific T cells is to engineer OVs to express TAAs, termed oncolytic vaccines (70). As such, TAAs are overexpressed in the tumor during viral replication, thus increasing the opportunity for immune responses to be generated toward tumor-specific antigens. However, successful antitumor activity has only been reported using model tumor antigens such as OVA or LacZ (71, 72) and the same approach was poorly effective against a self-TAA of low immunogenicity (70, 73). Altogether, these results suggest that overexpression of a TAA is insufficient to overcome immunosuppression in the tumor or immunodominant responses against viral antigens. Therefore, additional approaches are required to boost TAA-specific responses beyond these barriers. Indeed, significantly improved therapeutic efficacy can be achieved by adoptive transfer of TAA-specific transgenic T cells (74) or priming the host with a heterologous vector expressing the TAA (70), prior to oncolytic vaccination. Both approaches have been demonstrated to increase TAA-specific T cell frequency, by redirecting the focus of immune responses to the TAA, rather than the viral vector. Such OV-based cancer immunotherapies show promise by harnessing both oncolytic and antitumor immune-mediated attacks. Clinical evaluation of adoptive T cell transfer and OVs are currently underway as monotherapies (4, 75), however their success as a combination therapy has yet to be determined in human cancers.

## Challenges of Oncolytic Virus Monotherapy

Oncolytic viruses as a standalone therapeutic intervention have rarely been shown to induce complete, long-term regression of established tumors *in vivo* (76, 77). Tumors can develop multiple barriers to various anticancer therapies, including oncolytic virotherapy. Here, we detail several mechanisms that may hinder the therapeutic efficacy of OVs and the challenges they pose to the development of improved cancer virotherapies.

### Immunological barriers

The first line of defense against viral infection is the innate immune cells that patrol, detect, and rapidly eliminate foreign invaders. DCs express pattern recognition receptors that allow for the detection and subsequent uptake of viral particles. These activated DCs then migrate to draining lymph nodes to initiate the development of adaptive immune responses and to trigger NK cell activation. NK cells have a predominant role in impeding the early spread of viruses by directly lysing virally infected cells. Together, DCs and NK cells produce a range of cytokines that promotes T helper 1 (Th1) cell activity and potent cytotoxic T lymphocyte (CTL) responses that are necessary for clearing virus-infected cells (78). Additionally, humoral immune responses, namely the production of neutralizing antibodies by B cells and plasma cells, provide several lines of antiviral defense (79). Plasma cells derived from B1 cells imparts early defense against viral infection by producing polyspecific antibodies. CD4^+^ T helper cells then stimulate naive B cells at later stages, in order to generate memory B cells and long-lived plasma cells that produce high amounts of specific neutralizing IgG antibodies. Finally, the complement system, composed of soluble factors and cell surface receptors, blocks viral infection by acting on both the innate and adaptive immune responses. These mechanisms include, enhancing humoral immunity, regulating antibody effector mechanisms, and modulating T cell function (80).

Altogether, these immunological barriers pose a particular problem for repeat administration of OVs, by further promoting the development of adaptive antiviral immunity and reducing of its oncolytic effects. Moreover, a large fraction of the population has previously been exposed to the naturally occurring viruses that are commonly employed for generating therapeutic strains. Therefore, the infectious potential of recognized OVs (e.g., Ad, HSV) becomes limited by high levels of neutralizing antibodies (81, 82). These circulating antibodies can limit viruses from ever reaching the tumor site, especially since some viral particles, including HSV-1- and murine leukemia virus-derived viruses, are particularly prone to inactivation by the complement system (83, 84).

### Tumor environment

Tumors are a heterogeneous assortment of cells, composed of cancer cells, stromal cells, and infiltrating leukocytes, which promote tumor growth and maintain an immunosuppressive environment (85). Tumor-infiltrating leukocytes (TILs) can negatively regulate immune responses within the tumor, which include regulatory T cells (Tregs), myeloid derived suppressor cells (MDSC), and type 2 macrophages (M2). Their immunosuppressive functions can be exerted by secretion of cytokines (e.g., IL-10 and TGF-β), through inhibitory receptors (e.g., CTLA-4 and PD-L1) via cell contact, and secretion of amino-acid depleting enzymes (arginase and IDO) in the tumor microenvironment. Tumor cells themselves also have mechanisms to suppress antitumor immunity, such as the shedding of NKG2D ligands, MICA/B that blocks NK cell and T cell function (86) and facilitates the expansion of immunosuppressive CD4^+^ T cells (87). Soluble mediators released by tumor cells can directly inhibit CTLs, which include TGFβ, IL-10, PGE_2_, histamine, hydrogen peroxide, and adenosine (88), in addition to the hypoxic conditions and low extracellular pH that characterize the tumor environment (89, 90). Therefore, antitumor immune responses induced by modified-OVs may not be sufficient to combat a highly immunosuppressive tumor environment, unless additional therapeutic regimens are employed.

Preclinical and clinical evidence indicates that OVs often infect neoplastic lesions in a heterogeneous and incomplete fashion, irrespective of administration route and whether viruses are replication-competent or not (91–93). Physicochemical barriers to infection, including tumor size (94), the layers of dense intratumoral connective tissue (95), the elevated interstitial pressure (96), the poorly permissive vasculature (97), and the large areas of necrosis/calcification (98) play a prominent role in determining viral dissemination. As a result, oncolytic virotherapy may result in incomplete eradication of the primary tumor mass or possibly even promote metastasis of the tumor cells and eventually leading to recurrence of disease. Similar to what is observed in chemotherapy and radiotherapy regimens, malignant cells are also prone to become resistant to oncolytic virotherapy over time. This is presumably linked to the intrinsic nature of cancers to exhibit genomic instability and the propensity for accumulating mutations (99–101).

## Combining Immunomodulatory Chemotherapy with Oncolytic Virotherapy

Chemotherapeutic drugs used in combination with OVs can potentiate their cytotoxic mechanisms (9), but may also act to remove barriers to successful oncolytic virotherapy. Counteracting immunological barriers can improve the persistence of viruses and/or weaken the immunosuppressive forces within the tumor microenvironment. In this section, we summarize how pharmaceutical immunomodulators may be used to promote adaptive antitumor immune responses induced by OVs.

### Evading antiviral immune responses

Histone-deacetylase inhibitors (HDACi) are anti-inflammatory agents that can modulate immune responses to viral infection. By impeding the type I IFN response, a major component of the cellular innate antiviral response, HDACi’s can enhance the spread and antitumor effects of OVs (102). In addition, HDACi’s may also enhance OV efficacy through initial suppression of immune cell recruitment and inhibition of inflammatory cell pathways within NK cells (65). Similarly, a high throughput screen of pharmaceutical agents identified a novel drug (Vse1) that could enhance oncolytic virotherapy by disrupting the IFN-induced antiviral response and repressing antiviral gene transcripts (103). Another drug that can be used for immune suppression is cyclosphorine A, which markedly increased and prolonged the therapeutic effect of reovirus therapy of metastatic cancer (104, 105). However, the most common immunosuppressant drug used in the context of oncolytic virotherapy is cyclophosphamide (CPA); a chemotherapeutic alkylating agent that also induces apoptotic cell death. CPA has complex immune-modulating effects, affecting humoral and cellular mediators of both the innate and acquired immune responses. These immunosuppressive functions have been shown to enhance viral oncolysis and improve antitumor efficacy of HSV (83, 106, 107), Ad (108), measles virus (109), reovirus (110, 111), and VV (112). More specifically, at high doses, CPA has been shown to limit neutralizing antibody titers below the limit of detection during herpes virus hrR3 infection (106). Furthermore, *in vivo* depletion of complement significantly improved survival of HSV and CPA treated tumor-bearing rats (83). Global immunosuppression has also been reported to occur as a result of CPA therapy, including significant decreases in total white blood cell, lymphocyte, neutrophil, and monocyte counts in tumor-bearing mice. This was accompanied by significantly improved survival and decreased tumor volume in mice treated with both Ad and CPA relative to treatment with either therapy alone (108). Host lymphodepletion can enhance the therapeutic efficacy of OVs, as demonstrated by the reduction of antiviral antibody titers and subsequent promotion of viral persistence (113).

### Counteracting the immunosuppressive tumor environment

Regulatory T cells and MDSC are TIL populations that are a major component of the immunosuppressive tumor environment. Most pharmaceutical strategies that counteract immune resistance mechanisms within the tumor are aimed at depleting these inhibitory immune cell populations. Reduction of Tregs in cancer patients has been demonstrated to occur following treatment with fludarabine and paclitaxel (118, 119). Other chemotherapeutic drugs shown to decrease Tregs and inhibit their suppressive ability include CPA, paclitaxel, and temozolomide and cisplatin treatment, which enhances antigen-specific CD8^+^ T cells in murine tumor models (114–117). In particular, CPA, paclitaxel, and temozolomide can successfully reduce Treg activity (120–122) when delivered as metronomic doses (i.e., repetitive, low doses). In the case of CPA, metronomic doses serve to minimize toxicity and avoid global immunosuppression resulting from administering a single, high dose. Comparison of metronomic and maximum tolerated doses of CPA revealed that deletion of proliferating tumor-specific CTLs occurred in both dosing schedules. However, at metronomic doses, slower kinetics of deletion and survival of cells with a CD43^lo^ “memory” phenotype was observed, resulting in potent restimulatory capacity (122). This is supported by clinical evidence, in which metronomic CPA can deplete Tregs and restore T and NK cell effector function in advanced cancer patients (123). In the context of oncolytic virotherapy, preconditioning of mice with either CPA or anti-CD25 mAb to deplete Tregs enhances therapeutic benefits of oncolytic reovirus and VSV (111, 124). Furthermore, early clinical evaluation of metronomic CPA and oncolytic Ad combination treatment demonstrates improved antitumor efficacy, resulting from increased cytotoxic T cells and induced Th1 type immunity (125).

In healthy tissues, MDSCs play a protective role during inflammation to maintain homeostasis of pathogenic immune responses. However, accumulation of MDSCs in the tumor environment is also capable of promoting tumor growth by inhibiting antitumor effector T cell responses. They exert their effects through multiple immunomodulatory roles, such as upregulating the production of immune-suppressive factors (e.g., nitric oxide and reactive oxygen species), overexpressing anti-inflammatory cytokines (e.g., TGF-β and IL-10), suppressing proliferation and cytokine production by T cells and NK cells, and inducing apoptosis of CD8^+^ T cells (126). Furthermore, MDSCs can mediate the expansion of other immunosuppressive Treg and M2 populations (127–129). Numerous chemotherapeutic drugs have been used to deplete MDSCs, including gemcitabine, sunitinib, 5-FU, docetaxel, and retinoic acid (130–134). Combinations of OVs with various MDSC depleting drugs have been investigated at length, overall demonstrating improved survival in preclinical studies. The therapeutic benefits of using these OV-drug combinations depend on several factors, including the type of OV-drug combination used, the timing, frequency, and dosage of drug administration, and the cancer type targeted. However, given that these immunomodulatory drugs have other antitumoral effects, few studies have directly assessed their ability to deplete MDSCs in each context (135, 136). Notably, use of these drugs to deplete MDSCs can also positively or negatively affect oncolytic virotherapy. For instance, metronomic treatment of either gemcitabine or 5-Fu with oncolytic Ad, increases viral uptake by upregulating the expression of internalization receptors (137). Moreover, sunitinib negatively regulates the antiviral OAS-RNase L pathway, thus enhancing viral replication of VSV in tumors (138). In contrast, concurrent therapy of 5-Fu with HSV-1 inhibits virus replication and oncolysis (139). Therefore, optimization of these OV-drug combination strategies to benefit both the oncolytic and antitumor immune effects of OVs requires further investigation.

Given that chemotherapies have non-specific effects, some drugs can also modulate tumor cell immunogenicity to benefit oncolytic virotherapy. For example, paclitaxel can upregulate MHC class I expression and antigen-processing machinery components (140). 5^′^-aza-2^′^-deoxycytidine and 5-Fu have been shown to enhance tumor antigen expression (141–143), while Ara-C (cytosine arabinoside) treatment results in the induction of co-stimulatory molecules that provide a greater chance of effective immune activation (144, 145). Furthermore, both doxorubicin and Ara-C decreases the expression of immune checkpoint molecules, such as PD-L1, blocking their inhibitory effects on infiltrating T cells (146, 147). Some drugs, namely CPA, 5-Fu, and Dacarbazine, can sensitize tumor cells to CD8^+^ T cell-mediated apoptosis (148, 149), and thus may serve as ideal candidates for therapeutic combinations with various cancer immunotherapies.

## Evaluating the Landscape of OV-Drug Combinations

Tumor cell heterogeneity as a result of DNA instability promotes the natural selection of tumor progeny with greater proliferative capacity and invasive potential (150, 151). As a result, treatment methods that address a singular therapeutic strategy may be insufficient to completely eliminate tumor growth. OV-drug combinatorial strategies present countless different permutations, and consequently, numerous possibilities to mobilize multiple and simultaneous therapeutic approaches. However, previous studies that report synergistic outcomes from combining OVs with chemotherapy largely focus on a single therapeutic aspect, such as their effect on viral spread and persistence, cytotoxicity, or immunomodulation. As we become more familiar with how various chemotherapeutic drugs function, it is increasingly apparent that many drugs act in a multi-mechanistic fashion. In other words, chemotherapeutic agents can impact multiple biological processes, which in turn can further potentiate OV-drug interactions. For instance, rapamycin and its analogs have been shown to alter mTOR signaling to increase the tropism of OVs (152), inhibit angiogenesis (153), induce autophagy (32), and inhibit the function of M2 macrophages (154). HDACis such as Trichostatin A alter chromatin structure and regulate gene expression on an epigenetic level, leading to a wide range of biological effects like promoting tumor antigen presentation (155), improving tumor susceptibility to OVs (156–158), down-regulating the antiviral response (159), and targeting tumors and tumor vasculature (160). Lastly, receptor tyrosine kinase inhibitor sunitinib can down-regulate antiviral pathways (138), deplete MDSCs (131), inhibit M2 macrophages (161), and reduce tumor vascularization (162). Therefore, rather than evaluating individual therapeutic strategies that are complementary to oncolytic viral activity, combinatorial strategies using chemotherapeutic drugs should take into account of their entire functional repertoire, in order to determine the best overall approach. However, given the complex, interconnected biological pathways that regulate viral infection and tumor growth, assessing OV-drug combinations is not a simple task.

### Challenges of combination therapy

As previously mentioned, the biological pathways that OVs manipulate to support their replication are similar to those utilized by cancer cells to become increasingly malignant (e.g., defects in the IFN pathway, apoptotic-resistance, immune suppression). In fact, targeting certain pathways with chemotherapy will also, by association, compromise the replicative capacity of OVs. As a result, discernable conflicts between virus-enabled therapeutic strategies and drug-enabled therapeutic strategies may limit the extent to which the two can be combined. For example, viruses require actively dividing cells to maximize their replicative efficiency, while many anticancer agents are either cytotoxic or cytostatic with death-inducing or anti-proliferative effects, respectively (9). Furthermore, studies suggest that the leaky vasculature of tumors is exploited by viruses to successfully extravasate into the tumor site (163, 164). Some OVs can actually stimulate angiogenesis to increase vascular permeability in tumors (165). Thus, anti-angiogenic therapy may thus adversely affect the localization of OVs to the tumor microenvironment. Finally, modulation of the host immune response through chemotherapy may conflict with the therapeutic function of the oncolytic virus. For instance, low dose CPA may remove immunosuppressive cells such as Tregs to improve vaccine-induced adaptive antitumor immune responses; however, it also promotes the antiviral immune response, leading to early viral clearance (166). Conversely, high dose CPA may enhance viral oncolysis through wide-spread immunosuppression of the innate and adaptive antiviral immune response, but also completely abrogate the antitumor immune response (167). These conflicting mechanisms (apoptosis vs. viral replication, anti-angiogenesis vs. viral trafficking, antiviral immune responses vs. antitumor immune responses) are further compounded when we consider that drugs often regulate multiple biological host processes. Nevertheless, OV-drug combinations that demonstrate therapeutic incompatibility are still efficacious in some models. In these cases, it is likely that the number of beneficial interactions between OVs and drugs outweigh the number of detrimental effects, resulting in an overall enhanced therapeutic outcome. While current combinatorial strategies have been able to identify unique synergistic OV-drug platforms, the challenge going forward is to obtain a greater understanding of OV-drug interactions. Based on these exploratory findings, we will be able to identify optimal treatment conditions that minimize therapeutic trade-offs.

### Successful combination therapy is context-dependent

As previously mentioned, seemingly incompatible OV-drug combinations have shown therapeutic efficacy because their positive effects outweigh their negative effects. Based on these initial studies, it is also apparent that some factors can tip the OV-drug dynamic in favor of enhanced cancer therapy in one context, but also have the reverse effects in another. For instance, concurrent administration of 5-FU has been shown to inhibit the replication of wild-type HSV-1 strain KOS (139); however, the same drug has been shown to actually enhance viral replication of NV1066 (HSV-1 with a single copy of ICP0, ICP4, and γ_1_34.5 deleted) in pancreatic cancer cell lines (168). Interestingly, growth arrest and DNA damage as a result of 5-FU administration upregulates the expression of DNA damage-inducible protein GADD34, which bears significant homology with the deleted γ_1_34.5. As a consequence, GADD34 can functionally replace γ_1_34.5, prevent premature shutoff of protein synthesis, and thus enhance viral replication (169). Another factor that is demonstrated to be context-dependent is the schedule and dosage of drug delivery given during OV-drug combination therapy. However, if their costs and benefits to oncolytic virotherapy are clear, we may adjust these variables for an optimized therapeutic outcome. For example, VEGF blockade through a variety of small-molecule chemotherapeutics decreases the tumor uptake of systemic oncolytic HSV, but can actually improve the treatment of sarcoma-bearing mice if anti-angiogenic therapy is given subsequent to virus administration (170).

Overall, specific strategies to optimize OV-drug combinations depend on the circumstances of the model system. To this point, we have previously shown that systemic vaccination with recombinant VSV encoding the xenogeneic TAA, human dopachrome tautomerase (hDCT), was unable to induce robust tumor-specific immunity because the host immune response was predominantly redirected toward viral antigens expressed on the vector. Therefore, by adopting a heterologous prime-boost system whereby mice were initially primed with recombinant Ad-hDCT and boosted with VSV-hDCT, substantive immunity was generated against the tumor, while the antiviral response to VSV was dampened (70). The HDACi, MS-275, is an ideal candidate for combination therapy with this prime-boost system because it has previously been shown to decrease IFN responsiveness in tumors, thus augmenting viral oncolysis. However, MS-275 is also immunosuppressive and resulted in abrogation of the priming response if given concurrently with Ad-hDCT. Alternatively, if drug treatment was given concurrently with VSV-hDCT, the boosting response was unaffected and over 60% of mice challenged with intracranial melanoma were cured (171). Since MS-275 is an HDACi; an epigenetic modifier that can modify the expression of numerous genes, its range of effects have not yet been fully elucidated. As such, many unknown functional properties may still exist, especially in the context of oncolytic virotherapy.

## Concluding Remarks

War strategy dictates methods in which to arrange and maneuver military forces during armed conflicts. Using the available resources and landscape to your advantage is a key aspect to defeating the enemy. The analogy of OVs as fighters, “targeting” cancer cells and being “armed” with various genes, is commonplace in the literature. Its ability to induce antitumor immune responses is akin to the call for air support, bringing in additional fighters that can help to identify and target enemy forces. The introduction of chemotherapeutic drugs to the battlefield is then, chemical warfare; a wide-spread, indiscriminate weapon. With our various forces at hand, how do we determine the best strategy to defeat our opponents? As with any war strategy game, finding the best approach begins with knowing the enemy (type of cancer), knowing our forces (viruses, drugs, and immune cells), their strengths and weaknesses (function), and finally how they interact with each other on the battlefield (combination therapy). Before you make a move, you postulate various scenarios in which your opponent may attack, but also how you can take the advantage. In a similar fashion, to identify the most suitable approach to OV-drug combination therapies, we should adopt a broader perspective to the treatment of cancer. Then and only then, will we not only win some battles, but we may also win the war.

## Conflict of Interest Statement

The authors declare that the research was conducted in the absence of any commercial or financial relationships that could be construed as a potential conflict of interest.
